# Renal Stent Graft Migration Following Chimney Endovascular Aneurysm Repair

**DOI:** 10.3400/avd.cr.25-00034

**Published:** 2025-06-11

**Authors:** Shun-ichi Kawarai, Yuichi Ono

**Affiliations:** Department of Cardiovascular Surgery, Hachinohe City Hospital, Hachinohe, Aomori, Japan

**Keywords:** chimney endovascular aneurysm repair, stent graft migration, pararenal abdominal aortic aneurysm

## Abstract

A 78-year-old male presented with progressive enlargement of a pararenal abdominal aortic aneurysm following chimney endovascular aneurysm repair. The aneurysmal expansion was attributed to an endoleak secondary to migration of the left renal artery chimney stent graft, resulting in a 5-mm increase in aneurysm diameter over 6 months. Endovascular reintervention successfully induced aneurysm regression, with no recurrence of endoleak on annual imaging follow-up. While chimney endovascular aneurysm repair presents a minimally invasive alternative for managing complex aortic pathologies, including pararenal abdominal aortic aneurysms, vigilance regarding potential stent graft migration is essential.

## Introduction

Endovascular aneurysm repair (EVAR) has become the preferred intervention for abdominal aortic aneurysms (AAAs) due to its minimally invasive nature.^1)^ However, conventional EVAR poses challenges in treating complex aneurysms, such as pararenal AAAs, where an adequate infrarenal sealing zone is insufficient.^2,3)^ Chimney EVAR (ch-EVAR) has emerged as a viable alternative to open surgery for complex AAAs.^4–6)^ Nevertheless, geometric alterations in chimney stent grafts over time may necessitate secondary interventions.^7–9)^ Herein, we report a case of renal stent graft migration post-ch-EVAR, successfully managed via an endovascular approach.

## Case Report

**Figure 1:**
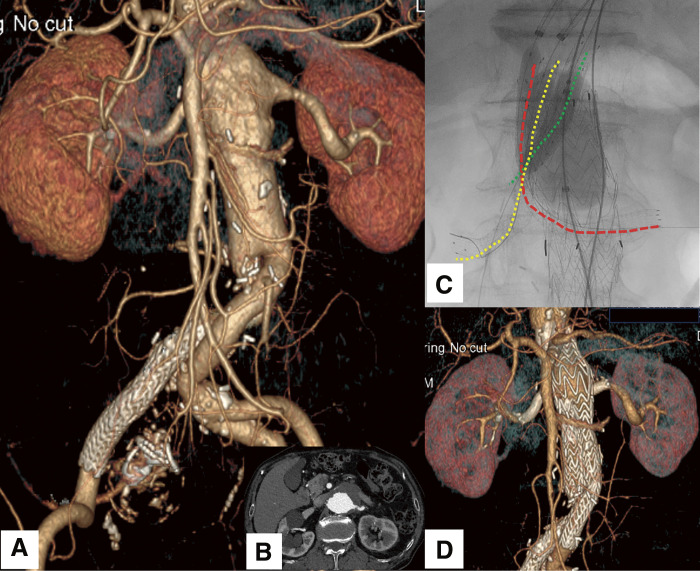
Fig. 1 (**A**) CTA reveals a pararenal AAA involving both renal arteries. (**B**) Axial imaging demonstrates a pararenal AAA. (**C**) During ch-EVAR, simultaneous kissing balloon technique is employed to minimize gutter-related endoleaks. The red dotted line delineates the displaced left renal artery chimney stent graft protruding into the aneurysm sac with an oblique trajectory. The yellow dotted line demarcates the right renal artery, while the green dotted line represents the SMA chimney stent graft. (**D**) At 6 months postoperatively, CTA confirms maintained patency of the chimney stent grafts and the absence of detectable endoleaks. ch-EVAR: chimney endovascular aneurysm repair; AAA: abdominal aortic aneurysm; CTA: computed tomography angiography; SMA: superior mesenteric artery

A 78-year-old male presented with progressive enlargement of a pararenal AAA following prior ch-EVAR. His medical history included open surgical repair for AAA a decade earlier and endovascular treatment for a right internal iliac aneurysm 4 years postoperatively. Owing to the progressive aneurysmal enlargement, which had reached a diameter of 57 mm, further intervention was imperative to avert the risk of rupture (**Figs. 1A** and **1B**). Considering the invasiveness of open surgery, an endovascular approach was desirable. To achieve an adequate proximal sealing zone, the patient underwent triple ch-EVAR to preserve perfusion to the superior mesenteric artery (SMA) and bilateral renal arteries (RAs). The aortic diameter at the level of the celiac artery origin (neck diameter) was measured at 27 mm, with a distance of 30 mm to the commencement of the aneurysmal segment. The diameters of the SMA and bilateral RAs were 7 and 6 mm, respectively. The main abdominal endograft utilized was a 35-mm Gore bifurcated Excluder endoprosthesis (W.L. Gore & Associates, Newark, DE, USA). Gore VIABAHN self-expanding stent grafts (W.L. Gore & Associates) were employed as chimney grafts. The SMA was revascularized using an 8-mm diameter, 5-cm-long stent graft, whereas the bilateral RAs were revascularized using 7-mm diameter, 10-cm-long stent grafts. During the procedure, the SMA and right RA were accessed via the right axillary artery, while the left RA was cannulated through the left axillary artery. The ch-EVAR was performed in a 3-step sequence. First, the main endograft was deployed and balloon-expanded in the standard fashion. Second, the chimney stent grafts were positioned and dilated using appropriately sized balloons. Finally, to minimize proximal endoleak resulting from gutter formation, a molding procedure involving simultaneous kissing balloon inflation of the main endograft and chimney stents was performed (**Fig. 1C**).

**Figure 2:**
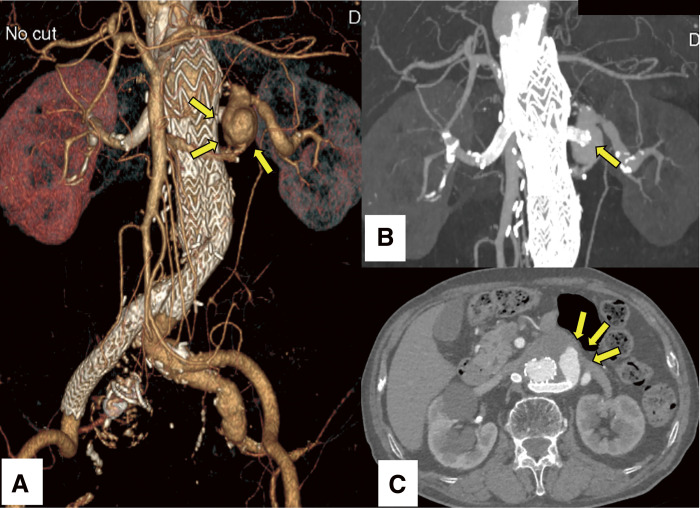
Fig. 2 (**A**) CTA demonstrates a significant endoleak within the aneurysmal sac (arrows). (**B**) MIP reconstruction imaging illustrates complete disengagement of the stent graft from the renal artery, with prolapse into the aneurysmal sac (arrow). (**C**) Axial imaging reveals a substantial endoleak within the aneurysmal sac (arrows), resulting in a 5-mm increase in sac diameter. MIP: maximum intensity projection; CTA: computed tomography angiography

The left renal chimney stent graft was posteriorly compressed by the main endograft, resulting in inflection into the aneurysmal sac with an oblique trajectory; however, completion angiography revealed no evidence of endoleak. At 6 months postoperatively, computed tomography angiography demonstrated complete aneurysm exclusion, with preserved patency of the chimney stent grafts and no discernible endoleak (**Fig. 1D**). Nevertheless, annual imaging surveillance identified a substantial endoleak within the aneurysmal sac (**Fig. 2A**), attributed to complete disengagement of the left renal chimney stent graft from the renal artery, followed by its migration into the aneurysmal sac (**Fig. 2B**). This substantial endoleak led to a 5-mm expansion in the diameter of the aneurysm sac (**Fig. 2C**).

Despite the absence of overt abdominal symptoms, the risk of aneurysmal rupture necessitated prompt interventional treatment. Open surgical repair was deemed high-risk due to its invasiveness and the potential for complications involving visceral vessels. Consequently, a minimally invasive endovascular approach was pursued. To mitigate further expansion, a bridging stent graft was required to re-establish continuity between the prolapsed chimney stent graft and the left renal artery.

**Figure 3:**
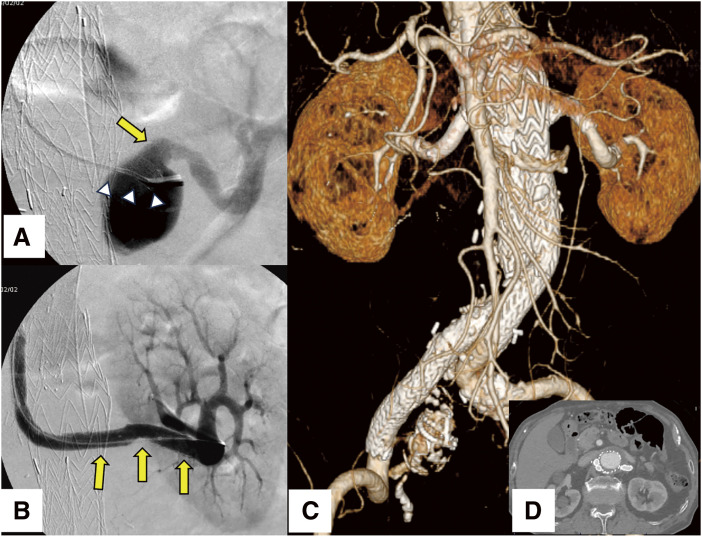
Fig. 3 (**A**) Angiography reveals a pronounced gap between the renal chimney stent graft (arrowheads) and the renal artery ostium (arrow), attributable to the enlarged aneurysmal sac. (**B**) Angiography confirms complete resolution of the endoleak following the deployment of a stent graft to bridge the gap between the chimney stent graft and the left renal artery (arrows). (**C**) CTA demonstrates no evidence of further stent graft migration or endoleak during a 3-year follow-up period. (**D**) Axial imaging demonstrates regression of the aneurysm. CTA: computed tomography angiography

The procedure was performed under general anesthesia. Surgical access was obtained via the left subclavian and left femoral arteries. To navigate the tortuous aortic arch, a pull-through technique was utilized to introduce a 12-Fr Gore Dry Seal sheath (W.L. Gore & Associates) from the left subclavian artery to the abdominal aorta. A 7-Fr guiding sheath (Parent Cross; Medikit, Tokyo, Japan) was advanced to the ostium of the left renal chimney stent graft. A 5-Fr catheter with curved tip (Medikit), in conjunction with a 0.035-inch hydrophilic guidewire (Radifocus; Terumo Medical, Tokyo, Japan), was inserted through the 7-Fr guiding sheath. After meticulous guidewire manipulation, successful cannulation of the displaced chimney stent graft was achieved. The 7-Fr guiding sheath was introduced into the left renal chimney stent graft smoothly. Due to the large aneurysmal sac and an extensive gap between the stent graft and the renal artery (**Fig. 3A**), prolonged guidewire maneuvering was required. After multiple catheter exchanges and precise wire manipulation, the guidewire was successfully inserted into the left renal artery. The guidewire was then substituted with a stiff type featuring a flexible tip (Amplatz Super Stiff; Boston Scientific Japan, Tokyo, Japan) via the 5-Fr catheter. Despite repeated attempts, the advancement of the 7-Fr guiding sheath into the renal artery proved futile due to bending within the aneurysm. Ultimately, a 7-mm-diameter, 59-mm-long balloon-expandable Gore VIABAHN VBX stent graft was deployed directly to bridge the chimney stent graft and the left renal artery. After balloon dilatation, post-deployment angiography confirmed complete resolution of the endoleak (**Fig. 3B**). The patient was discharged without complications, and subsequent follow-up imaging confirmed sustained aneurysm regression without stent migration or recurrence of endoleak (**Figs. 3C** and **3D**).

## Discussion

Advancements in vascular surgery have significantly improved the safety profile of AAA management. EVAR, offering reduced perioperative morbidity, has become the preferred modality for treating anatomically suitable aneurysms. Studies by Schmitz-Rixen et al. report a 30-day mortality of 1.16% for EVAR, compared to 3.17% for open aneurysm repair (OAR), reinforcing the preference for endovascular approaches in AAA management. However, long-term durability remains a concern, with higher rates of reintervention following EVAR relative to OAR.^1)^

Pararenal AAA, characterized by aneurysmal extension to the visceral aorta, poses additional challenges due to an inadequate proximal landing zone. Open surgical repair requires suprarenal and/or supramesenteric clamping and reconstruction, which is very invasive, with substantial operative mortality and morbidity. While fenestrated and branched EVAR offers a promising solution, its applicability is limited by the need for patient-specific customization, prolonged manufacturing times, and unavailability in certain regions, such as Japan.^2,3)^

EVAR combined with the concomitant chimney/snorkel technique represents an alternative endovascular therapeutic approach for branch revascularization in complex aortic pathologies, including pararenal AAAs, and has garnered increasing recognition. This technique was first reported in the literature in 2003. Lachat et al. reported a 99% technical success and a 98% target vessel patency rate in their application of the chimney and periscope technique for complex aortic aneurysms over a 2-year follow-up period. Additionally, nearly 95% of patients exhibited either a reduction or stabilization in aneurysm size on imaging.^4)^ Donas et al. analyzed data from the PERICLES registry, encompassing 517 patients treated with ch-EVAR, and reported a 30-day mortality of 3.7% in elective cases during a mean follow-up period of 17 months. Their findings support ch-EVAR as a viable, off-the-shelf, and readily available alternative for managing complex aortic pathologies, including pararenal AAAs.^4,5)^ Taneva et al. presented their analysis of the PERICLES registry, providing the previously lacking long-term data on the ch-EVAR technique. The results demonstrated favorable outcomes, with over 50% of patients surviving beyond 5 years and a chimney graft patency rate of 92%. Their analysis identified the absence of an infrarenal neck and the use of a sealing zone with a diameter exceeding 30 mm as risk factors for type IA endoleak and chimney stent graft patency loss. The primary objective of ch-EVAR is to ensure that an adequate amount of abdominal endograft material encases the chimney stents, thereby minimizing the formation of gutters. They emphasized the critical importance of achieving a proximal sealing zone of at least 20 mm and employing an abdominal endograft with approximately 30% oversizing. In our case, the application of the chimney technique enabled the establishment of a sufficient proximal sealing zone. By utilizing a 35-mm main body graft, corresponding to an oversizing ratio of approximately 30%, the chimney stent grafts were effectively enveloped by the endograft material to mitigate gutter-related endoleaks.^6)^

Nevertheless, stent graft migration remains a critical concern. Morphometric analyses indicate that chimney grafts are susceptible to geometric alterations over time.^7)^ Hahtapornsawan et al. observed that the renal arteries underwent cephalad displacement, whereas the corresponding chimney grafts migrated caudally following ch-EVAR. Chimney grafts commonly adopt an oblique rather than a parallel configuration.^8)^ Tran et al. further elucidated that chimney graft orientation influences endoleak risk, particularly when renal chimney stent grafts traverse >90° from their ostium, assuming an oblique trajectory.^9)^ In our case, the left renal chimney stent graft was displaced posteriorly at approximately 150°, rendering it susceptible to migration under pulsatile flow dynamics. To mitigate the risk of chimney stent graft displacement, steerable sheaths may serve as essential tools for enhancing the alignment of renovisceral branch stent grafts during ch-EVAR procedures.^9)^ The self-expanding and the balloon-expandable stent grafts demonstrate comparable outcomes, serving as safe and effective bridging stent options during EVAR for the treatment of complex aortic pathologies.^10)^

Following reintervention, no further migration or endoleak was observed over a 3-year follow-up period. These findings underscore the necessity of long-term surveillance in patients undergoing ch-EVAR, particularly those with obliquely oriented chimney grafts.

## Conclusion

We report a case of renal stent graft migration following ch-EVAR, successfully managed via endovascular intervention. While ch-EVAR remains a viable alternative for high-risk patients with pararenal AAA, the risk of stent graft migration necessitates ongoing imaging surveillance to ensure long-term procedural success.

## Declarations

### Informed consent

Informed consent was obtained from the patient to publish this case report.

### Ethical approval

We obtained approval from the institutional ethics committee of Hachinohe City Hospital (Approval number: 2502).

### Disclosure statement

The authors declare that they have no conflict of interest.

### Author contributions

Study conception: SK

Data collection: all authors

Analysis: SK

Investigation: all authors

Critical review and revision: all authors

Final approval of the article: all authors

Accountability for all aspects of the work: all authors.
